# Redescription of *Potamonautes sidneyi* (Rathbun, 1904) (Decapoda, Potamonautidae) and description of a new congeneric species from KwaZulu-Natal, South Africa

**DOI:** 10.3897/zookeys.657.11623

**Published:** 2017-02-16

**Authors:** Nasreen Peer, Gavin Gouws, Eric Lazo-Wasem, Renzo Perissinotto, Nelson A.F. Miranda

**Affiliations:** 1 DST/NRF Research Chair in Shallow Water Ecosystems, Nelson Mandela Metropolitan University, PO Box 77000, Port Elizabeth 6031, South Africa; 2 South African Institute for Aquatic Biodiversity (SAIAB), Private Bag 1015, Grahamstown, 6140, South Africa; 3 Division of Invertebrate Zoology, Peabody Museum of Natural History, Yale University, PO Box 208118, New Haven, CT 06520-8118, USA

**Keywords:** Taxonomy, Brachyura, freshwater, morphometrics, KwaZulu-Natal

## Abstract

A new species of freshwater crab, *Potamonautes danielsi* **sp. n.**, is described from the southern region of the KwaZulu-Natal Province, South Africa. *Potamonautes danielsi* most closely resembles *Potamonautes sidneyi* which is re-described here, but can be distinguished by a suite of key morphological characters including carapace shape and width, slim pereopods, inflated propodi of the chelipeds, and the shape and terminal segment length:subterminal segment length ratio of the 1^st^ gonopod. In a previous study (Gouws et al. 2015), a 9.2–11.8 % divergence was found in the mitochondrial COI and 16S genes of the *Potamonautes sidneyi* clade, allowing for the delineation of a new species. Despite the clear molecular distinction between the two species, it is difficult to separate them based on individual morphological characters, as there is a great deal of overlap even among key features. The new species is found in slow-moving mountain streams and pools at high altitudes between Umhlanga and Mtamvuna, in KwaZulu-Natal.

## Introduction

*Potamonautes sidneyi* (Rathbun, 1904) was first described from “Natal, southern Africa” (presently KwaZulu-Natal Province, South Africa). The original description was sourced from the Muséum National d’Histoire Naturelle, Paris, allowing us to locate the syntypes. Two type specimens are known and are lodged at the Peabody Museum, Yale University (original catalogue number 1191). These were collected by Sarah Abraham in 1871, but no accurate locality data were provided. Rathbun (1904) listed another individual, collected from Port Natal (presently Durban), as *Potamonautes sidneyi*, but pointed out certain differences from the types, notably the lack of a concave ridge behind the eyes. Although the description (Rathbun, 1904) highlighted the main difference between *Potamonautes sidneyi* and *Potamonautes perlatus* (H. Milne-Edwards, 1837), it was brief and relatively vague with few quantifiable or measurable distinctions between the two species.

*Potamonautes sidneyi* is regarded as one of the most widespread potamonautid species, occurring from the eastern parts of South Africa, northwards to Zimbabwe and Malawi (Gouws et al. 2002). Within the province of KwaZulu-Natal (KZN), *Potamonautes sidneyi* was thought to occur in the low-lying midlands regions from the Drakensberg to the coast and to inhabit the entire coastal zone from the Maputaland (northern KZN) to the northern border of Pondoland (northern Eastern Cape ending at the southern KZN border) (Gouws and Stewart 2001, Gouws et al. 2015). However, recent genetic analyses have shown that these KwaZulu-Natal populations include two distinct genetic lineages, i.e. a northern lineage and a southern lineage, with the divergences warranting recognition of these lineages as separate species (Gouws et al. 2015). The issue of which lineage corresponds to the described *Potamonautes sidneyi* was difficult to resolve due to various morphological similarities between the two lineages, the vagaries of the original species description and the lack of a precise locality for the type material (Rathbun 1904).

Examination of high resolution photographs of key diagnostic features, including the carapace, chelipeds and male gonopods of *Potamonautes sidneyi* type specimens alongside specimens collected from both lineages revealed that specimens from the northern Maputaland lineage likely represent *Potamonautes sidneyi* s. str. as they match the type specimens in terms of the following: the slim propodi of the chelipeds, the stout pereopods, the shape and terminal segment length:subterminal segment length ratio of the 1^st^ gonopod; and its larger size. The southern lineage, thus, represents a new species and is described in this paper by NP and GG while RP and NAFM contributed to the information on its ecology and natural history, and EL-W contributed to the redescription of *Potamonautes sidneyi*. The delineation of a new species at the northern border of the Pondoland region is significant, based on the distribution of the lineages revealed by Gouws et al. (2015), as it interrupts the unclear and often confusing transition between *Potamonautes sidneyi* and *Potamonautes perlatus* at locations where the two species overlap and are often morphologically indistinct (Barnard 1950, Gouws and Stewart 2001).

## Materials and methods

### Collection of crabs

Detailed photos of the original syntype and additional specimens of *Potamonautes sidneyi* were obtained from the Invertebrate Zoology Division at the Yale Peabody Museum (CT, USA) and the Muséum National d’Histoire Naturelle (Paris, France) respectively.

**Figure 1.**
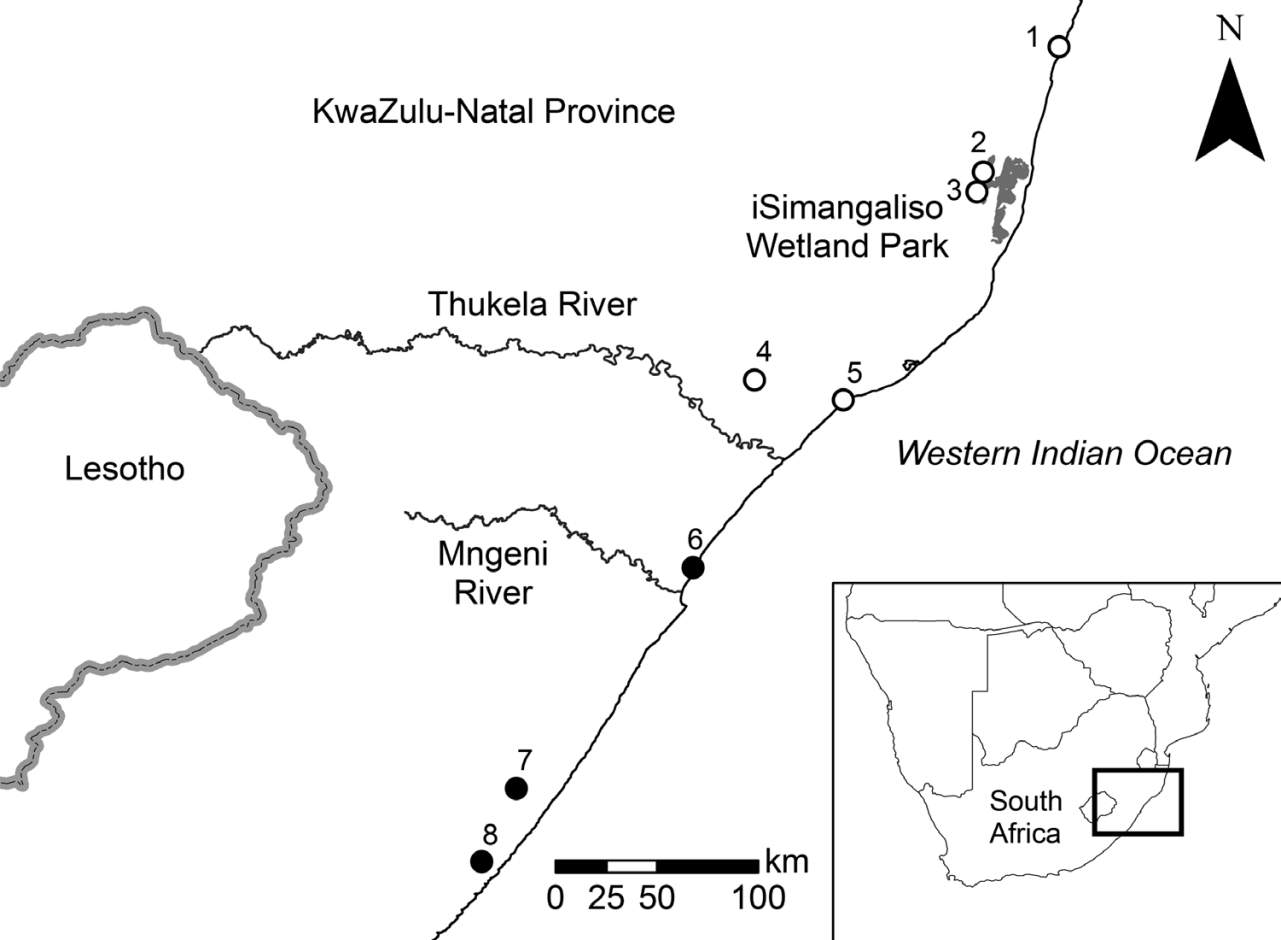
Sampling localities for the present study of *Potamonautes* in KwaZulu-Natal Province on the east of South Africa. The collection localities of *Potamonautes danielsi* sp. n. are indicated by black dots, while those of *Potamonautes sidneyi* are indicated by white open dots. Sites are indicated by numbers which correspond to localities as follows: (Site 1) Lake Sibaya, (Site 2) Mpophomeni Stream, (Site 3) Hluhluwe, (Site 4) Entumeni, (Site 5) Siyayi, (Site 6) Mhlanga, (Site 7) Oribi Gorge and (Site 8) Mtamvuna. Map modified from Gouws et al. (2015; copyright Gouws et al. 2015; licensee: AOSIS Publishing).

Crab specimens were collected from various localities around KwaZulu-Natal (Fig. 1). For the taxonomic description and morphometric analyses, crabs from localities 1 (Lake Sibaya), 2 (Mpophomeni Stream), 3 (Hluhluwe), 5 (Siyayi), 6 (Mhlanga), 7 (Oribi Gorge), and 8 (Mtamvuna) were used. The crabs from Mtamvuna, Mhlanga and Oribi Gorge were used to describe the new species, while the morphology and morphometric analyses of *Potamonautes sidneyi* were conducted using the syntype specimens, as well as crabs from Lake Sibaya, Mpophomeni Stream, Hluhluwe and Entumeni. Crabs were collected by hand or by net and preserved in 70% ethanol. Photographs were taken using a Canon Powershot G12 digital camera.

### Morphological and morphometric analyses

For examination of *Potamonautes danielsi* type specimens, a pair of Vernier callipers was used to measure morphological variables. A Nikon SMZ25 microscope fitted with a Nikon Digital Sight DS-Fi2 camera was used for macro-examination and to take photos of gonopods and mouthparts. A Canon Powershot G12 was used to photograph the carapace and appendages.

For the redescription of *Potamonautes sidneyi* *s. str.*, a lectotype was designated from the syntypes housed at the Peabody Museum.

Abbreviations for repositories and provinces:

YPM  Yale Peabody Museum, New Haven, Connecticut, United States of America


SAM Iziko South African Museum, Cape Town, South Africa


AM Albany Museum, Grahamstown, South Africa


DNM Ditsong National Museum of Natural History, Pretoria, South Africa


NMMU  Nelson Mandela Metropolitan University, Port Elizabeth, South Africa


MNHN Muséum National d’Histoire Naturelle


EC  Eastern Cape Province, South Africa


WC  Western Cape Province, South Africa


KZN  KwaZulu–Natal Province, South Africa


Abbreviations for all morphological and morphometric characters (following Gouws et al. 2001):

CL  Carapace length;


CWW  Carapace widest width;


CWP  Carapace posterior width;


PFCD  Distance between postfrontal crest and anterior margin;


ED  Distance between orbits;


CWA  Distance between exorbital teeth;


CH  Carapace height;


AW6  Width of sixth abdominal segment;


MCPL  Major cheliped propodus length;


MCPH  Major cheliped propodus height;


P2ML  Pereopod 2, merus length;


P2MW  Pereopod 2, merus width;


s2/s3  First sternal groove (suture between the second and third sulci);


s3/s4  Second sternal groove (suture between the third and fourth sulci);


CRDL  Right cheliped, dactyl length;


CLDL  Left cheliped, dactyl length;


CRPL  Right cheliped, propodus length;


CRPW  Right cheliped, propodus width.


Eight variables, including six carapace variables and dimensions of the propodus of the right cheliped (CL, CWW, CH, PFCD, ED, CWA, CRPW and CRPL), were log transformed and used to statistically analyse morphometric differences between the two species, by means of a discriminant functions analysis in STATISTICA v12.5 (Statsoft Inc., Tulsa, OK, USA; www.statsoft.com). Classification functions were calculated and individuals were then reassigned to groups based on *a*-*priori* probabilities. Canonical scores were plotted for both species as a frequency histogram to examine distinctions between the two forms. Lastly, linear regression analyses were used to examine variation among the two species for combinations of specific variables.

## Taxonomic description

### Suborder Brachyura Linnaeus, 1758

#### Superfamily Potamoidea Ortmann, 1896

##### Family Potamonautidae Bott, 1970

###### Subfamily Potamonautinae Bott, 1970

####### Genus *Potamonautes* Macleay, 1838

######## Potamonautes sidneyi

Taxon classificationAnimaliaDecapodaPotamonautidae

(Rathbun, 1904)

Table 1 ; Figs 4 , 5 , 6A

Potamon (Potamonautes) perlata var. a Krauss 1843: 37
Potamon (Potamonautes) sidneyi Rathbun, 1904: plate 14, fig. 3.-Rathbun, 1905: 163-166.-Stebbing 1910: 295.-Lenz 1912: 7.-Barnard 1935: 438, fig 1c.-Chace 1942: 222. Barnard 1950: 184, 187, fig 34b.-Chace 1953: 440.-Cumberlidge 1998: 204.
Potamonautes (Orthopotamonautes) sidneyi Bott 1955: 278-279, 235, fig 46, pl XX, figs 1a–d.
Thelphusa perlata Milne Edwards 1837: 13.-Kingsley 1880: 36
Thelphusa corrugata Heller 1865: 32, pl IV, fig 1.-Milne-Edwards 1869: 181.

######### Type series.

Lectotype: male, CL = 35.6 mm, CWW = 52.4 mm (Table 1), Port Natal, 1871, S. Abraham legit (YPM IZ 001191).

Paralectotype: CL = 27 mm, CWW = 36 mm, Port Natal, 1871, S. Abraham legit (YPM IZ 078196)

**Table 1.** Ranges of measurements (mm) for 12 morphometric variables of the *Potamonautes danielsi* sp. n. holotype and paratypes collected from Mtamvuna, Oribi Gorge and Mhlanga, as well as *Potamonautes sidneyi* (Rathbun, 1904) specimens collected from Lake Sibaya, Hluhluwe, Mpophomeni Stream and Entumeni.

**Variable**	***Potamonautes danielsi* sp. n.**			***Potamonautes sidneyi***	
	**Holotype**	**Males (n= 14)**	**Females (n= 22)**	**Males (n=12)**	**Females (n= 10)**
CL	18.5	12.4–23.6	10.4–34.3	13.64–34.2	13.3–41.6
CWW	25.8	16.1–34.0	13.2–45.5	19.11–43.5	19.1–54.8
CWP	12.8	9.2–15.2	7.4–23.1	9.97–21.3	10.4 - 29
PFCD	2.6	1.4–3.0	1.3–4.7	2.3–4.2	1.9–5.5
ED	8.9	6.0–10.5	5.7–15.8	7.8–16.4	7.4–19.52
CWA	18.8	13.2–24.4	11.5–26.5	16.5–33.1	15.9–39.6
CH	9.3	5.7–12.5	5.3–13.4	7.0–16.0	6.9–20.3
AW6	5.4	1.1–6.9	4.6–24.0	4.1–12.6	5.1–32.2
MCPL	16.6	7.6–33.1	7.8–22.3	11.3–29.6	11.3–34.0
MCPH	9.41	4.81–19.5	2.9–9.7	4.3–12.8	4.4–16.0
P2ML	10.29	6.1–15.7	5.6–14.5	8.0–16.7	7.9–20.5
P2MW	4.25	2.1–6.5	2.3–6.2	3.4–7.2	3.3–8.7

**Figure 2.**
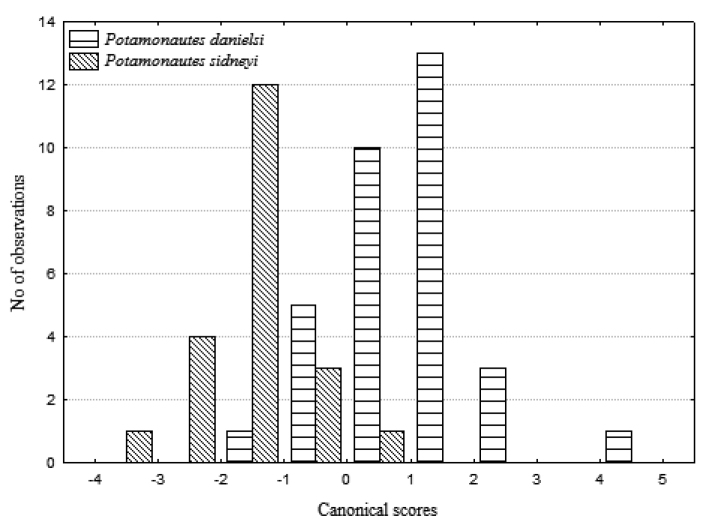
Histogram of canonical scores for *Potamonautes danielsi* sp. n. and *Potamonautes sidneyi* calculated from a discriminant function analysis, based on six carapace and two right cheliped variables.

**Figure 3.**
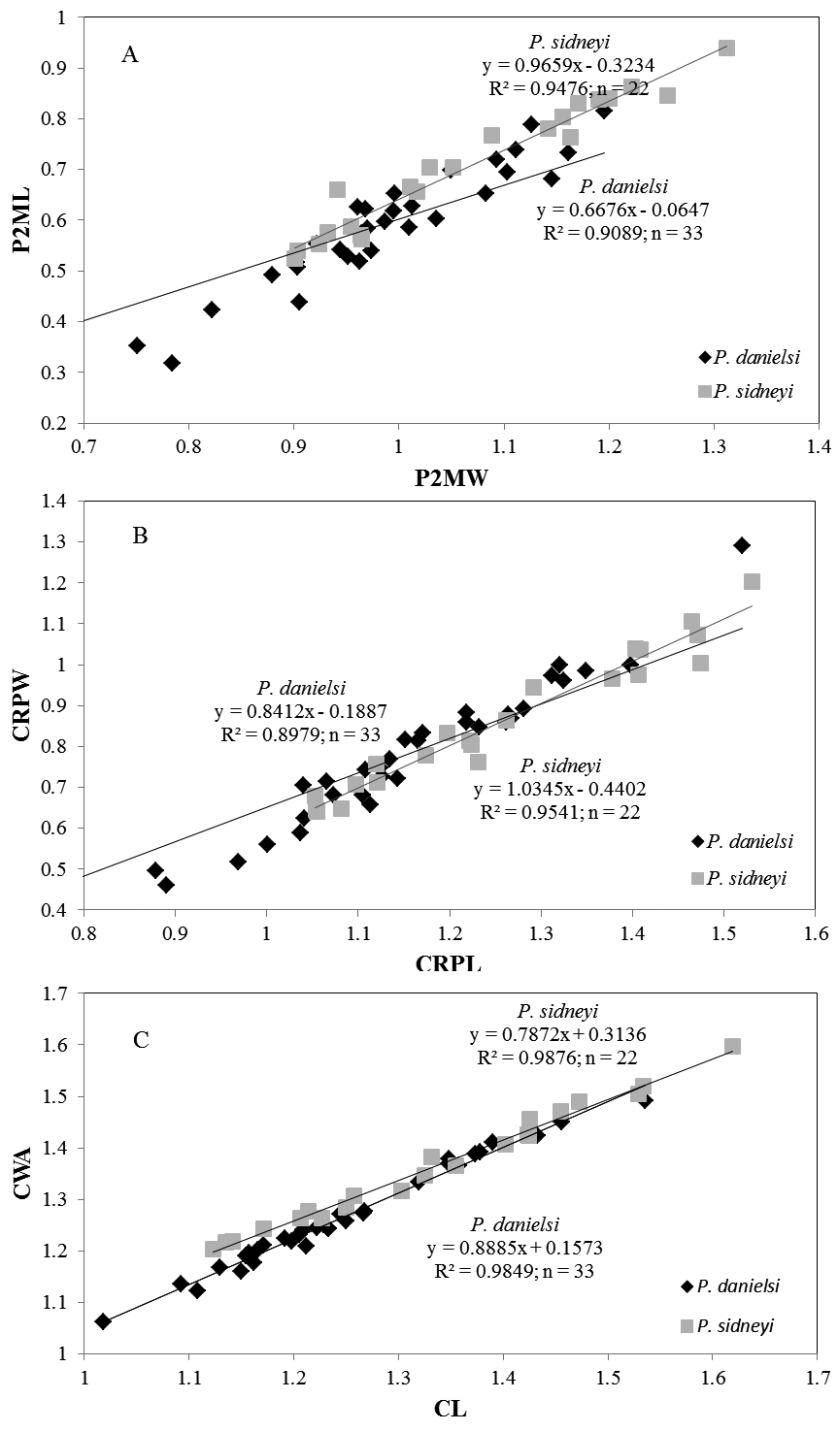
Regression analyses of morphometric measurements of *Potamonautes danielsi* sp. n. and *Potamonautes sidneyi*. Regression analyses of: **A** LogP2ML over LogP2MW **B** LogCRPW over LogCRPL; and **C** LogCWA over LogCL between the two species, *Potamonautes danielsi* sp. n. and *Potamonautes sidneyi*. All differences between regressions were statistically significant (p < 0.001).

######### Additional material examined.

Locality unknown, MNHN B3841 (MNHN-IU-2000-3841) (1♀), CWW = 50 mm; Ikhamanzi River, SAM A43967 (2 ♂, 2 ♀), CWW = 45 mm (♂), 61 mm (♀), 29°16'13"S, 30°38'30"E; Mseleni River Bridge near Lake Sibaya, SAM A41112 (2 ♂, 2 ♀), CWW = 50 mm (♂), 50 mm (♀), 27°21'50"S, 32°31'35"E, coll. M. Coke on 19 Aug 1997; Linwood (Kwa Gqishi Stream near Midmar Dam), SAM A43960 (1 ♀), CWW = 36 mm, 29°33'50"S, 30°05'40"E, coll. O. Bourquin on 4 May 1998; Pinetown (Durban), SAM A41139 (2 ♂, 2 ♀), CWW = 45 mm (♂), 70 mm (♀); Mtunzini (Otungulu Pan, Umlalazi Nature Reserve), coll. B. Stewart and P. Cook on 10 Apr 1992; SAM A41171 (1 ♀), CWW = 35 mm, 29°03'17"S, 31°39'52"E, coll. M. Coke and A. Wood on 08 Mar 1994; Mdumbeni River, SAM A41979 (1 ♂), CWW = 42 mm, 28°58'07"S, 30°22'20"E, coll. M. Coke on 10 Jan 1995; Mvudi River (University of Venda), SAM A41993 (1 ♂), CWW = 37 mm, 22°55'S 30°30'E, coll. B.C.W. van de Waal on 06 Mar 1994; Phongolo River (Rivierplaats, Luneburg), SAM A41966 (1 ♂, 1 ♀), CWW = 39 mm (♂), 39 mm (♀), 27°21'20"S, 30°27'17"E, coll. M. Coke and S. de Jager on 19 Jan 1995; Mariti River (The Gums), SAM A41131 (1 ♀), CWW = 37 mm, 24°56'18"S, 31°04'43"E, coll. D. Weeks and V. Makunyane on 17 Aug 1992; Hlimbitwa River (Klipnek near Hermannsburg), SAM A43965 (1 ♂), CWW = 41 mm, 28°57'56"S, 30°45'20"E, coll. M. Coke on 20 Dec 1996; Syzygium swamp forest (University of Zululand), SAM A43937 (1 ♂, 1 ♀), CWW = 33 mm (♂), 40 mm (♀), 28°51'25"S, 31°51'02"E, coll. B.A Stewart, P.A Cook, P.E. Reavell, L. Hoenson and G. Gouws on 24 Jan 1997; Bilanyoni River (Koppie Alleen near Luneburg), SAM A41959 (1 ♂, 1 ♀), CWW = 43 mm (♂), 42 mm (♀), 27°17'25"S, 30°34'55"E, coll. M. Coke on 19 Jan 1995; Manzibomvu River (Upper reaches, Hluhluwe Game Reserve), SAM A43935 (1 ♂, 1 ♀), CWW = 53 mm (♂), 50 mm (♀), 28°02'20"S, 32°05'10"E, coll. B.A Stewart, L. Hoenson and G. Gouws on 28 Jan 1997; Shinane River (Tributary of Mutshindini, Venda), SAM A41994 (1 ♂, 1 ♀), CWW = 27 mm (♂), 40 mm (♀), 22°53'S 30°31'E, coll. B.C.W. van de Waal on 25 Oct 1995; Stream behind dunes at Amatikulu Hatchery, SAM A41104 (1 ♂, 1 ♀), CWW = 36 mm (♂), 36 mm (♀), 29°04'30"S, 31°38'35"E, coll. M. Coke on 10 Mar 1994; Palmiet River (Westville, Durban), SAM A41114 (1 ♀), CWW = 37 mm, 29°49'53"S, 30°54'37"E, coll. M. Coke and D. Coutts on 10 Feb 1994; Wekeweke River (plunge pool, Nshongweni), SAM A43948 (1 ♀), CWW = 35 mm, 29°50'07"S, 30°43'20"E, coll. M. Coke and J. Craigie on 22 May 1997; Nsuze River (R614 Bridge, Fawnleas, Tongaat Road), SAM A43969 (1 ♂), CWW = 33 mm, 29°22'28"S, 30°56'25"E, coll. M. Coke and M. Peters on 06 Feb 1996; Mhlathuze River (D223 Bridge near Babanango), SAM A43976 (1 ♀), CWW = 60 mm, 28°28'46"S, 31°04'09"E, coll. M. Coke and M. Peters on 14 June 1996; Blood River (at laager behind monument), SAM A43941 (1 ♀), CWW = 71 mm, 28°06'15"S, 30°32'40"E, coll. T. Ridgway and G. Gouws on 16 May 1997; Amagoda River (outside Vryheid), SAM A43939 (1 ♂, 1 ♀), CWW = 52 mm (♂), 56 mm (♀), 27°46'45"S, 30°46'07"E, coll. T. Ridgway and G. Gouws on 15/16 Oct 1997; Mhulumbela River (Onverwacht picnic site, Itala Game Reserve), SAM A43949 (1 ♂, 1 ♀), CWW = 40 mm (♂), 44 mm (♀), 27°32'00"S, 31°19'02"E, coll. M. Coke on 06 Nov 1997; Ifaye River (Mount Elias, Fawnleas), SAM A43968 (1 ♀), CWW = 40 mm, 29°19'12"S, 30°46'53"E, coll. M. Coke and M. Protheroe on 09 Oct 1995; Macabuzela Stream (near Dakaneni, Hluhluwe Game Reserve), SAM A43942 (1 ♂, 1 ♀), CWW = 56 mm (♂), 40 mm (♀), 28°02'32"S, 32°09'51"E, coll. T. Ridgway and G. Gouws on 19 May 1997; Mvuzane River (D50 Bridge), SAM A43977 (1 ♂, 1 ♀), CWW = 45 mm (♂), 45 mm (♀), 28°49'41"S, 31°11'23"E, coll. Coke, Eckard and Louw on 20 Aug 1997; Mahai Stream (Royal Natal National Park), SAM A41311 (1 ♀), CWW = 37 mm, 28°41'15"S, 28°56'30"E, coll. E. Dickson on 04 Dec 1994; Mgeni River (Albert Falls Dam), SAM A43963 (1 ♂), CWW = 36 mm, 29°26'47"S, 30°20'50"E, coll. M. Coke and M. Peters on 15 May 1996; Bilanyoni River (D27 Bridge, Luneburg), SAM A41965 (1 ♂, 1 ♀), CWW = 47 mm (♂), 45 mm (♀), 27°19'20"S, 30°38'00"E, coll. M. Coke on 19 Jan 1995; Crocodile Rover (Rietvlei Farm, AM GEN256J (2 ♂), CWW = 28 mm, 25°22'49"S, 30°33'03"E, coll. unknown on 21 Nov 1959; Inyamvubu River (5km above Craigieburn), AM GEN843 (1 ♀), CWW = 64 mm (♀), 29°11'24"S, 30°16'12"E, coll. M. Coke and P. Couldon on 12 Apr 1989; Mkomaas River (Nhlavini Stream, Coothill Farm), AM GEN847 (1 ♂, 1 ♀), CWW = 40 mm (♂), 35 mm (♀), 30°11'24"S, 30°09'00"E, coll. C. Arter and M. Coke on 03 Oct 1988; Nontshibongo River (below Gala Forest), AM GEN848 (1 ♂, 1 ♀), CWW = 37 mm (♀), 29°59'24"S, 29°48'36"E, coll. C. Arter and M. coke on 05 Oct 1988; KwaCota River (The Springs Farm), AM GEN849 (1 ♀), CWW = 31 mm (♀), 30°04'48"S, 29°52'12"E, coll. C. Arter and M. Coke on 07 Oct 1988; Manzana River (Tributary at Rondspring Farm), AM GEN953 (1 ♂, 1 ♀), CWW = 73 mm (♂), 27°33'00"S, 31°00'36"E, coll. M. Coke on 23 Apr 1991; Nosonto River Headwaters (near Vryheid), AM GEN954 (1 ♂, 1 ♀), CWW = 57 mm (♂), 35 mm (♀), 27°44'24"S, 30°37'48"E, coll. M. Coke on 21 Mar 1991; Grantleighspruit (near Mooi River), AM GEN955 (1 ♂, 1 ♀), CWW = 57 mm (♀), 29°10'48"S, 29°58'48"E, coll. M. Coke on 25 Apr 1991; Lynspruit (Waterhoek Farm, Vryheid), AM GEN956 (1 ♂, 1 ♀), CWW = 36 mm (♂), 27°44'24"S, 30°36'36"E, coll. M. Coke on 21 Mar 1991; Bells Spruit (Ladysmith), AM GEN957 (1 ♂, 1 ♀), CWW = 37 mm (♂), 32 mm (♀), 28°32'24"S, 29°48'00"E, coll. M. Coke on 20 Mar 1991; Hlambizandla Stream (Gluckstad), AM GEN959 (2 ♂, 1 ♀), CWW = 57 mm (♂), 70 mm (♀), 27°58'48"S, 31°03'36"E, coll. M. Coke on 22 Mar 1991; KwaMbizankulu River (KwaMbizankulu Stream, Bevenson Farm), AM GEN960 (1 ♂, 1 ♀), CWW = 37 mm (♂), 36 mm (♀), 27°59'24"S, 31°07'12"E, coll. M. Coke on 22 Mar 1991; Bivane River (Frischgewaagd Farm), AM GEN962 (1 ♂, 1 ♀), CWW = 27 mm (♀), 27°32'24"S, 30°47'24"E, coll. M. Coke and J. van Niekerk on 24 Mar 1991; Bivane River (Kruger Bridge), AM GEN963 (1 ♂, 1 ♀), CWW = 28 mm (♂), 46 mm (♀), 27°31'12"S, 30°49'12"E, coll. M. Coke and J. van Niekerk on 24 Mar 1991; Bridal Veil Falls (near Sabie), AM GEN141A (1 ♂, 1 ♀), CWW = 48 mm (♂), 25°04'48"S, 30°44'24"E, coll. unknown on 08 Jul 1959; Lake Sibaya (North bank, South basin, East shore), AM SIB46H (2 ♂, 1 ♀), CWW = 36 mm (♂), 29 mm (♀), 27°23'24"S, 32°42'36"E, coll. B. Allanson on 18 Jan 1967; Waterpoort (Salt pan), DNM TM5061 (1 ♀), CWW = 36 mm, 22°53'50"S, 29°37'44"E; Lake Fundusi, DNM TM5063 (1 ♂), CWW = 33 mm (♂), 22°51'04"S, 30°18'35"E. Wakkerstroom, DNM TM5015 (1 ♀), CWW = 41 mm, 27°21'23"S, 30°07'47"E, collected by P. Simons and G. van Dam; Wakkerstroom, DNM TM5041 (1 ♀), CWW = 45 mm, 27°21'23"S, 30°07'47"E,Ratomba, DNM TM5183 (1 ♂), CWW = 48 mm, 23°04'00"S, 30°09'59"E.

######### Diagnosis.

Carapace flat and scabrous. Anterolateral margin heavily granulated. Postfrontal crest complete bearing concavity behind orbital regions. Propodi of chelipeds straight and slim. Pereopods 2-5 stout. Gonopod 1 displaying high terminal segment length:subterminal segment length ratio of 0.31.

######### Description of lectotype.

**Figure 4.**
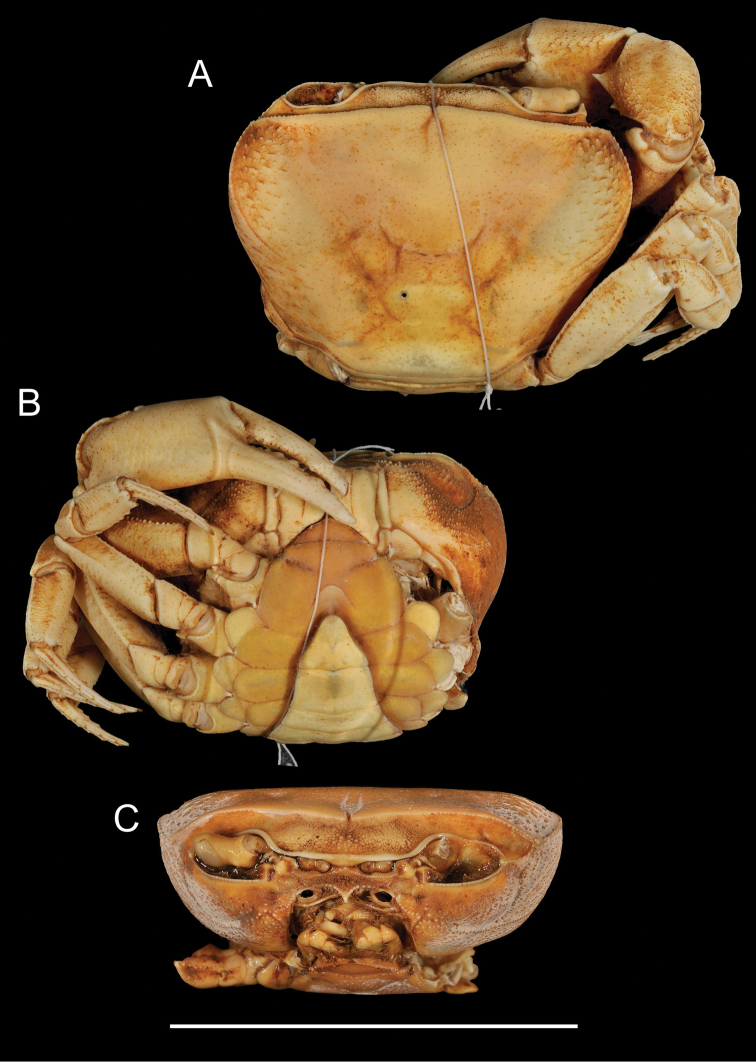
*Potamonautes sidneyi* (Rathbun, 1904). Male holotype CWW 47 mm (Yale Peabody Museum catalogue number 1191) **A** dorsal view **B** ventral view, and **C** cephalothorax, frontal aspect. Scale bar: 50 mm. Photos: Eric Lazo-Wasem.

*Carapace* (Figs 4A, C, 9A). Cephalothorax flat (CH/CL = 0.54), wide (CWW/CL =1.33), ovoid in frontal aspect. Branchial region flat forming angle with anterolateral margin. Anterior margin straight bearing concavity behind orbital regions, heavily granulated. Epigastric lobes poorly defined above postfrontal crest; two slight indentations present, forked from midpoint of postfrontal crest. Postfrontal crest heavily granulated, straight and distinct from epibranchial region to midpoint, curving downward at epibranchial region. Exorbital teeth present; no epibranchial teeth present, anterolateral margin serrated. Flank of carapace scabrous, well-defined epimeral sutures dividing pterygostomial region from subhepatic and suborbital regions, well-defined pleural groove dividing subhepatic region from suborbital region.

*Sternites* (Fig. 4B). Sternites 1 and 2 fused, first sulcus (s1/s2) absent. Second sulcus (s2/s3) prominent, running completely across sternum, third sulcus (s3/s4) projecting downwards medially to abdominopelvic region. Sulci and episternal sulci thereafter well-defined but shallow.

**Figure 5.**
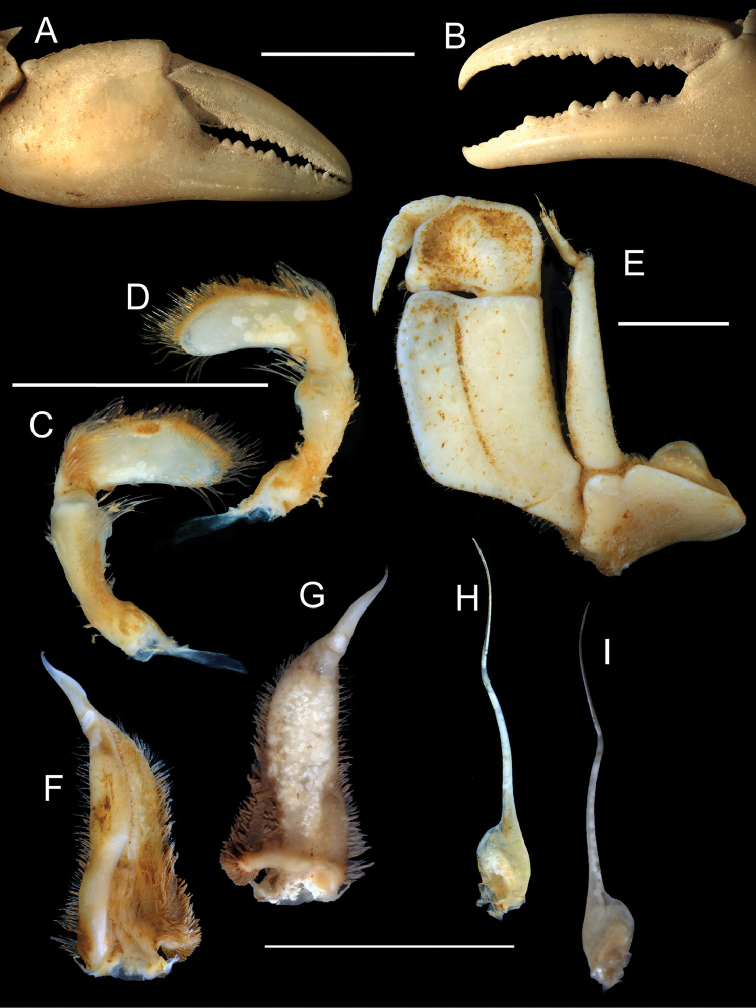
*Potamonautes sidneyi* (Rathbun, 1904). male holotype CWW 47 mm (Yale Peabody Museum catalogue number 1191) **A** major cheliped **B** minor cheliped **C** right mandibular palp posterior view **D** right mandibular palp anterior view **E** 3^rd^ maxilliped **F** left gonopod 1 anterior view **G** left gonopod 1 posterior view **H** left gonopod 2 anterior view, and **I** left gonopod 2 posterior view. Scale bars: 5 mm (**A, B**), 5 mm (**C, D**), 5 mm (**E**), 10 mm (**F, G, H, I**). Photos: Eric Lazo-Wasem.

*Third maxillipeds* (Figs 4C, 5E). Filling entire buccal frame except for oval respiratory openings medially above maxilliped. Ischium scabrous, with wide groove running vertically. Flagellum on exopod very long, straight.

*Mandibular palp* (Fig. 5C, D). Consisting of two segments; terminal segment smooth and undivided, with hirsute margins; dense tuft of long setae arising from base. Subterminal segment bulbous in appearance.

*Pereopods* (Figs 4A, B, 5A, B). No substantial heterochely (CRDL/CLDL > 0.91. CRDL broken at the tip. Refer to fig. 4B). Dactyl of major cheliped slightly arched. Small slim interspace formed when closed in minor chelipeds; not possible to establish if same applies to major cheliped due to broken tip. Propodus slim (CRPW/CRPL = 0.40), exhibiting ~21 cutting teeth. Carpus on either side containing one prominent tooth followed by one small tooth. Meri granulated with strong medial tubercle on inner lateral face. Pereopods 2 (ML/MW = 2.29) and 5 (ML/MW = 2.39) moderately stout; pereopod 3 longest among pereopods; pereopod 5 shortest. Ventral margins of meri smooth; ventral margins of propodi serrated; dactyli serrated, ending in sharp points.

**Figure 6.**
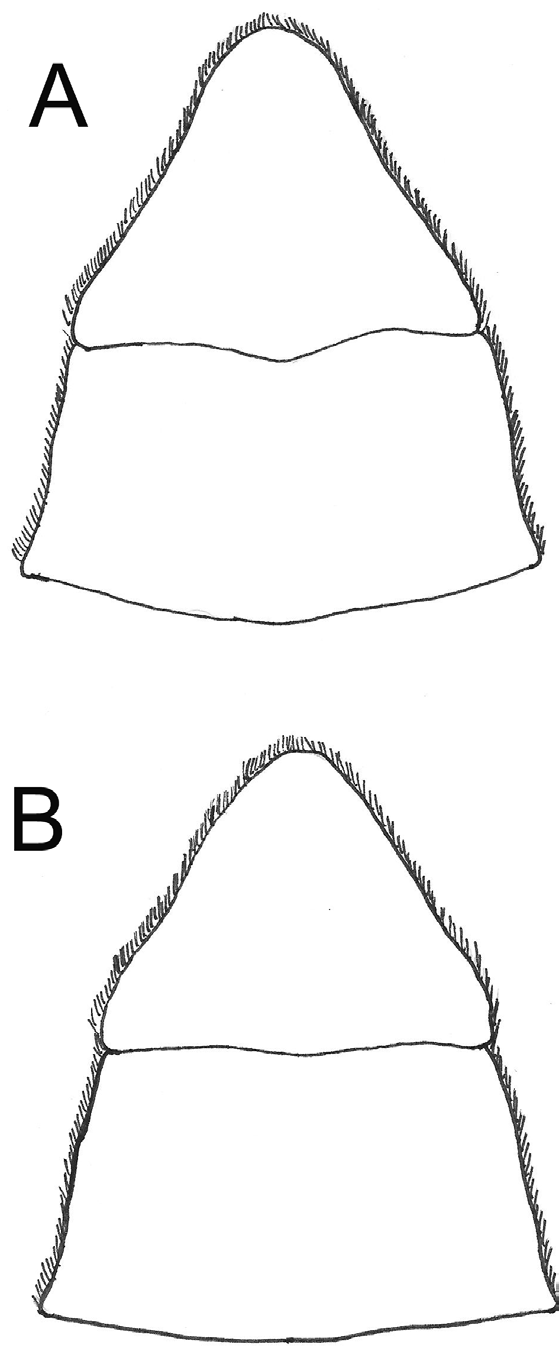
Morphological comparison of somites 5 and 6 between **A** *Potamonautes sidneyi* (Rathbun, 1904) and **B** *Potamonautes danielsi* sp. n.

*Pleon* (Figs 4B, 6A). Somites 1-6 four-sided with distally-rounded triangular terminal somite (telson). First 5 somites broad and short; somite 6 longer, about 1.7 times as wide as long, distal margins concave, lateral margins slanted towards medial line, swelling slightly at articulation with somites 5 and 7; telson terminally rounded, lateral margins concave, swell at articulation with somite 6; hirsute lateral margins.

*Pleopods* (Fig. 5F, G, H, I). Gonopod 1 widest at base; both subterminal and terminal segments tapering, ending with sharp point. Medial margin fairly straight displaying extrusion near base; lateral margin concave relative to midline; both margins hirsute. Groove extending almost entire length of gonopod, visible on dorsal surface, lined with setae. Gonopod 1 terminal segment long (0.31 times length of subterminal segment), curving outwards (i.e. away from midline) when viewed dorsally. Gonopod 2 consisting of two segments. Distal segment very long (0.57 times length of basal segment), slim; basal segment with wide elongated base sharply becoming narrow around 0.4 of length; narrow process forming at this point leading to distal segment.

**Variation**. The species appears to be extremely variable, with the northern populations displaying a more pronounced scabrosity and granulation on the chelipeds and carapace with fine hairs in some cases. Pereopods range from slender to stout. The inflation of the propodi on the chelipeds varies too, with some specimens bearing slim chelipeds while others possess more inflated propodi similar to that of *Potamonautes danielsi*.

**Live colouration**. Usually a variety of brown, ranging from chocolate brown to light brown or beige. In some cases pereopods are lighter than the carapace itself, a feature seen more in northern populations.

######### Distribution.

Known to extend from Lake Sibaya in the north to Durban along the coast (Fig. 1), based on recent sampling and Gouws et al. (2015). This species has also been recorded in the Mpumalanga Province.

######### Type locality.

South Africa, Port Natal. Collected by Mme Sarah Abraham. The specimens were received by the Peabody Museum (Yale) in 1871, along with other material. There is a possibility that the crab was among material accumulated for years, before it was sent to Yale and therefore it is uncertain as to whether the specimens were actually collected in 1871. While Mme Abraham was known to reside in Maphumulo (approximately 55 km north of Port Natal), it is unknown where exactly the specimens were from.

######### Etymology.

The species was named by Miss M.J. Rathbun in honour of Professor Sidney I. Smith from Yale University.

######### Remarks.

Only two type specimens were indicated in the original description, i.e. one male and one female from Port Natal both reposited at the Yale Peabody Museum. The lectotype was chosen on the basis of its designation as the only male type specimen in the original description. Compared to the lectotype, the paralectotype appears to be the same on the basis of the carapace scabrosity and granulation, the stout pereopods and the slim propodus of the cheliped. Although two additional specimens were mentioned, they were not designated as types. The first, a single female belonging to the Muséum National d’Histoire Naturelle, was uncertainly classified as *Thelphusa corrugata* Heller (Milne-Edwards, 1869). Following examination, it resembles *Potamonautes sidneyi* *s. str.* based on the stout limbs and slender propodi of the chelipeds. Despite the wide distribution and lack of specific locality all three specimens represent the same species i.e. *Potamonautes sidneyi* *s. str.*

######## Potamonautes danielsi

Taxon classificationAnimaliaDecapodaPotamonautidae

Peer & Gouws sp. n.

http://zoobank.org/D1C2B8F4-6903-4BC6-8798-36BF711B6033

######### Type series.

Holotype: male, CL = 18.5 mm (Table 1), mountain stream running into the Mtamvuna River, Mtamvuna Nature Reserve (31°03'31.60"S, 30°10'26.11"E; elevation 140 m), 18 November 2015, N. Miranda and N. Peer legit (SAMC A83487).

Allotype: female, CL = 22.8 mm (Table 1), collection details as per holotype (SAMC A83488).

Paratypes: (Table 1) collection data same as above, SAMC A83489 (1 ♂, 1 ♀); collection data same as above, NMMU (2 ♀); Umhlanga Nature Reserve, SAMC A83490 (1 ♂, 1 ♀), 29°42'13"S, 31°05'27"E, 28 January 2013, N. Miranda and N. Peer legit; Oribi Gorge Nature Reserve, SAMC A83491 (2 ♂), 30°40'55"S, 30°18'26"E, 30 January 2013, N. Peer and J. Raw legit. Mvoti River (Makabeleni), SAM A43970 (1 ♂), CWW = 53 mm, 29°14'45"S, 30°56'15"E, coll. M. Coke on 09 Nov 1995; Pongola Bush Nature Reserve, SAM A41984 (1 ♂, 1 ♀), CWW = 46 mm (♂), 47 mm (♀), 27°19'40"S, 30°29'15"E, coll. M. Coke and M. Protheroe on 05 Apr 1995; Mvoti River (‘Canema’ below falls), SAM A43966 (1 ♂, 1 ♀), CWW = 36 mm (♂), 51 mm (♀), 29°10'00"S, 30°41'15"E, coll. M. Coke and M. Peters on 02 May 1996; Mtamvuna Nature Reserve, SAM A44982 (1 ♂), CWW = 40 mm, 31°02.704'S 30°10.080'E, coll. S. van Noort on 10 Nov 2000; Nwaku River (D356 Causeway near Nwaku Store), SAM A43974 (2 ♂, 1 ♀), CWW = 69 mm (♂), 55 mm (♀), 28°56'35"S, 31°23'50"E, coll. M. Coke on 19 Aug 1997; Umhlanga Nature Reserve, SAM A41179 (1 ♀), CWW = 41 mm, 29°42'40"S, 31°05'35"E, coll. M. Coke on 04 Aug 1994; Pandana River (Schuilhoek, near Luneburg), SAM A41976 (1 ♂), CWW = 30 mm, 27°23'22"S, 30°30'55"E, coll. M. Coke on 20 jan 1995; Giant’s Castle (Path to Bannerman’s Hut), SAM A44178 (1 ♀), coll. M. Hamer on 27 Mar 1995; CWW = 35 mm, Spartelspruit (above D251 Bridge), SAM A43952 (1 ♂), CWW = 62 mm, 27°50'02"S, 30°32'45"E, coll. M. Coke on 07 Sep 1997; Pandana River (Welbedacht, near Luneburg), SAM A41970 (1 ♂, 1 ♀), CWW = 52 mm (♂), 42 mm (♀), 27°22'40"S, 30°34'30"E, coll. M. Coke on 20 Jan 1995; Nuwejaarspruit tributary (Oliviershoek Pass summit, near waterfall, Drifters Inn), SAM A43944 (1 ♂, 1 ♀), CWW = 39 mm (♂), 40 mm (♀), 28°33'25"S, 29°03'21"E, coll. B.A Stewart, P.A Cook, l. Hoenson and G. Gouws on 21 Jan 1997; Mfongosi (Zululand), SAM A41100 (1 ♂, 1 ♀), CWW = 37 mm (♂), 45 mm (♀),coll. W.C. Jones on May 1918; Mzinto River (Esperanza Bridge, near Umzinto), SAM A43946 (1 ♀), CWW = 40 mm, 30°20'28"S, 30°58'50"E, coll. Coke and Murray on 14 Jan 1998; Mgeni River (causeway near Nagle Dam), SAM A41968 (2 ♂), CWW = 65, 51 mm, 29°39'10"S, 30°41'10"E, coll. M. Coke and J. Craigie on 11 May 1995; Phongolo River (Rivierplaats, Luneburg), SAM A41969 (1 ♂, 1 ♀), CWW = 49 mm (♂), 55 mm (♀), 27°21'10"S, 30°27'13"E, coll. M. Coke on 19 Jan 1995; Greytown pond, SAM A41972 (1 ♂), CWW = 53 mm, 29°04'03"S, 30°35'25"E, coll. M. Coke on 10 Jan 1995; Mlaas River (Maybole, near Baynesfield), SAM A41971 (1 ♂, 1 ♀), CWW = 40 mm (♂), 41 mm (♀), 29°44'20"S, 30°15'22"E, coll. M. Coke on 07 Mar 1995; Tsakwe River (Protest, near Kempslust), SAM A41962 (1 ♂), CWW = 38 mm, 27°27'00"S, 30°31'15"E, coll. M. Coke on 20 Jan 1995; Mbango River (Port Shepstone), SAM A43945 (1 ♀), CWW = 44 mm, 30°45'05"S, 30°26'40"E, coll. M. Coke on 14 Mar 1996; Umgababa River (below dam wall), SAM A43947 (1 ♀), CWW = 59 mm, 30°08'52"S, 30°48'52"E, coll. J. Craigie on 02 Oct 1997; Inzimuke River (Utrecht), SAM A43953 (1 ♂), CWW = 33 mm, 27°37'47"S, 30°21'35"E, coll. Coke and Murray on 26 Jan 1998; Upper Bouthosloop (near Mount Carmel), AM GEN246A (1 ♂, 1 ♀), CWW = 35 mm (♂), 25°24'00"S, 30°43'48"E, coll. unknown on 21 Nov 1959; Nkonzo and Mzimkulu Rivers (Underbush Farm, near Creighton), AM GEN837 (2 ♂), CWW = 39, 50 mm, 29°58'12"S, 29°48'36"E, coll. C. Arter on 04 Oct 1988; Nkonzo River (at Nxumeni River confluence), AM GEN842 (1 ♂, 1 ♀), CWW = 46 mm (♂), 29°58'48"S, 29°51'00"E, coll. C. Arter on 04 Oct 1988; Mgeweni River (at Mgeni River confluence), AM GEN908 (1 ♂), CWW = 47 mm, 29°39'36"S, 30°40'48"E, coll. C. Dickens and M. Coke on 04 Jan 1991.

######### Diagnosis.

*Potamonautes danielsi* exhibits smooth to lightly granulated carapace flanks and epibranchial regions compared to those of *Potamonautes sidneyi* *s. str.* which often display a pronounced scabrosity and heavy granulation. The anterolateral margin is straight and complete. *Potamonautes danielsi* has long slender pereopods and the propodi of the chelipeds are inflated in contrast to the stout pereopods and slim propodi of *Potamonautes sidneyi* *s. str.* *Potamonautes danielsi* has a low terminal segment length:subterminal segment length ratio of gonopod 1 compared to that of *Potamonautes sidneyi* *s. str.*

######### Description of holotype.

**Figure 7.**
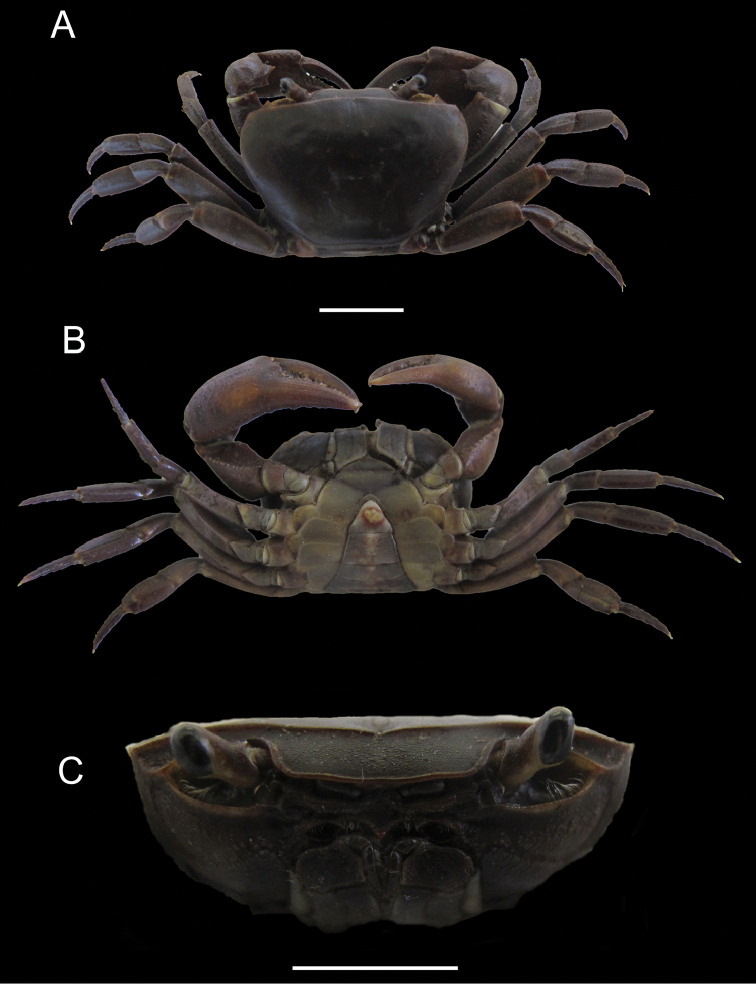
*Potamonautes danielsi* sp. n. male holotype CWW 25.8 mm (SAMC A83487) **A** dorsal view, **B** ventral view, and **C** cephalothorax, frontal aspect. Scale bar: 10 mm. Photos: Nasreen Peer.

*Carapace* (Figs 7A, C, 9B). Cephalothorax flat (CH/CL = 0.49), wide (CWW/CL = 1.34), almost rectangular in frontal aspect. Branchial region flat forming angle with anterolateral margin. Anterior margin straight, smooth with occasional faint granulation. Urogastric, cervical and intestinal grooves well-defined; cardiac and branchial grooves well-defined where attached to urogastric and cervical grooves, becoming poorly defined and faint towards edge of carapace. Epigastric lobes poorly defined above postfrontal crest; two slight indentations present, forked from midpoint of postfrontal crest. Postfrontal crest slightly granulated at branchial region, straight and distinct from epibranchial region to midpoint, curving downward at epibranchial region. Exorbital teeth present; no epibranchial teeth present. Flank of carapace scabrous, well-defined epimeral sutures dividing pterygostomial region from subhepatic and suborbital regions, well-defined pleural groove dividing subhepatic region from suborbital region.

*Sternites* (Fig. 7B). Sternites 1 and 2 fused, first sulcus (s1/s2) absent. Second sulcus (s2/s3) prominent, running completely across sternum, third sulcus (s3/s4) projecting downwards medially to abdominopelvic region. Sulci and episternal sulci thereafter well-defined but shallow.

*Third maxillipeds* (Fig. 7C, 8E). Filling entire buccal frame except for oval respiratory openings medially above maxilliped. Ischium scabrous, with wide groove running vertically. Flagellum on exopod very long, curved to form a loop.

**Figure 8.**
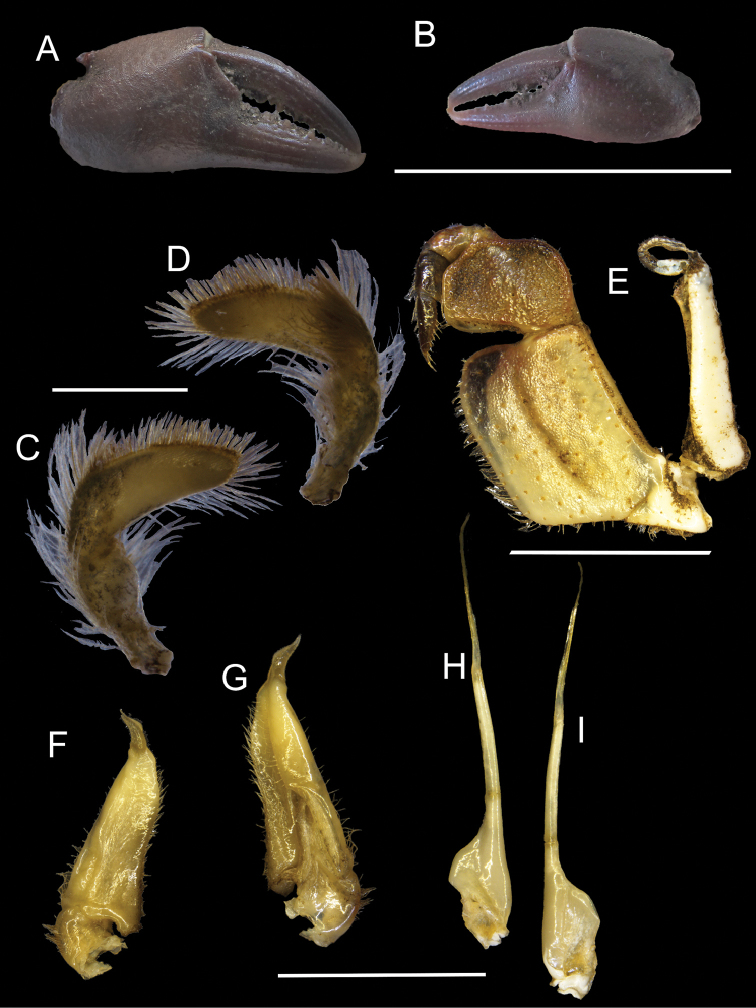
*Potamonautes danielsi* sp. n. male holotype CWW 25.8 mm (SAMC A83487). **A** major cheliped **B** minor cheliped **C** right mandibular palp posterior view **D** right mandibular palp anterior view **E** 3^rd^ maxilliped **F** left gonopod 1 anterior view **G** left gonopod 1 posterior view **H** left gonopod 2 anterior view, and **I** left gonopod 2 posterior view. Scale bars: 10 mm (**A, B**), 2 mm (**C, D**), 5 mm (**E**), 5 mm (**F,G, H, I**). Photos: Nasreen Peer.

*Mandibular palp* (Fig. 8C, D). Consisting of two segments; terminal segment smooth and undivided, with hirsute margins; dense tuft of setae emerging from base. Subterminal segment enlarged distally then compressed at joint with terminal segment.

*Pereopods* (Figs 7A, B, 8A, B). No substantial heterochely (CRDL/CLDL = 1.04). Dactyl of major cheliped slightly arched; small slim interspace formed when closed in major and minor chelipeds. Twenty-five cutting teeth present on dactyl of major cheliped; 4 larger and more prominent than the rest. Propodus inflated (CRPW/CRPL = 0.46), exhibiting 20 cutting teeth. Carpus on either side containing one prominent tooth followed by one small tooth. Meri strongly granulated at margins. Pereopods 2 (ML/MW = 2.59) and 5 (ML/MW = 2.66) moderately slender; pereopods 3 and 4 equal in length and longest among pereopods; pereopod 5 shortest. Ventral margins of meri smooth; ventral margins of propodi serrated; dorsal margins of meri and propodi bearing fine bristles; dactyli serrated, ending in sharp points.

*Pleon* (Figs 6B, 7B). Somites 1-6 four-sided with distally-rounded triangular terminal somite (telson). First 5 somites broad and short; somite 6 longer, about 1.6 times as wide as long, distal margins straight or slightly concave, lateral margins slanted towards medial line, lateral margins swell slightly at articulation with somite 5; telson terminally rounded, lateral margins concave, swell at articulation with somite 6; hirsute lateral margins

*Pleopods* (Fig. 8F, G, H, I). Gonopod 1 widest at base; both subterminal and terminal segments tapering, ending with sharp point. Medial margin fairly straight; lateral margin concave relative to midline; both margins hirsute. Groove extending to almost entire length of gonopod, visible on dorsal surface, lined with setae. Gonopod 1 terminal segment short (0.21 times length of subterminal segment), curving away from midline when viewed dorsally. Gonopod 2 consisting of two segments. Distal segment very long (0.67 times length of basal segment), slim; basal segment with wide elongated base sharply becoming narrow around 0.4 of length; narrow process forming at this point leading to distal segment. Gonopod 2 fairly straight, barely curving outward when viewed ventrally; curving slightly inward towards medial line at tips of distal segment.

**Variation.** The species appears to be extremely variable, with the northernmost Mhlanga population more closely resembling *Potamonautes sidneyi* *s. str.* rather than the *Potamonautes danielsi* sp. n. holotype. The epibranchial corners of the Mhlanga type are scabrous and granulated. Granulation, however, is not as pronounced as in *Potamonautes sidneyi* *s. str.* and no fine hairs are observed on the carapace. The terminal segments of both gonopods in the Mhlanga type are also more curved (typical of *Potamonautes sidneyi* *s. str.*), as opposed to straight (typical of *Potamonautes danielsi*). The flagellum on the exopod of the third maxilliped is highly variable. In the Mtamvuna population, the flagellum is long and curves backward to form a loop in some specimens. In both the Oribi and Mhlanga populations this is not seen. Instead, the flagellum curves upwards, similar to the pattern observed in most other potamonautid species.

**Live colouration.** Variable. Carapace colour ranges from purple to reddish-brown to greenish-brown. Carapace and pereopods are fairly uniform in colour with tips of dactyli and chelipeds usually displaying a lighter orange colour.

######### Distribution.

Currently known to extend from Mhlanga (Durban North) to the Mtamvuna River on the northern border of Pondoland (southern KZN), based on recent sampling and the results published in Gouws et al. (2015). Morphological examination of museum specimens shows that this species is also present in the Mpumalanga Province.

######### Holotype locality.

South Africa, KwaZulu-Natal: Mtamvuna Nature Reserve (31°00'23"S, 30°09'12"E).

######### Etymology.

The species is named after Professor Savel Daniels in recognition of his valuable contribution to knowledge of freshwater crabs in southern Africa.

######### Remarks.

**Figure 9.**
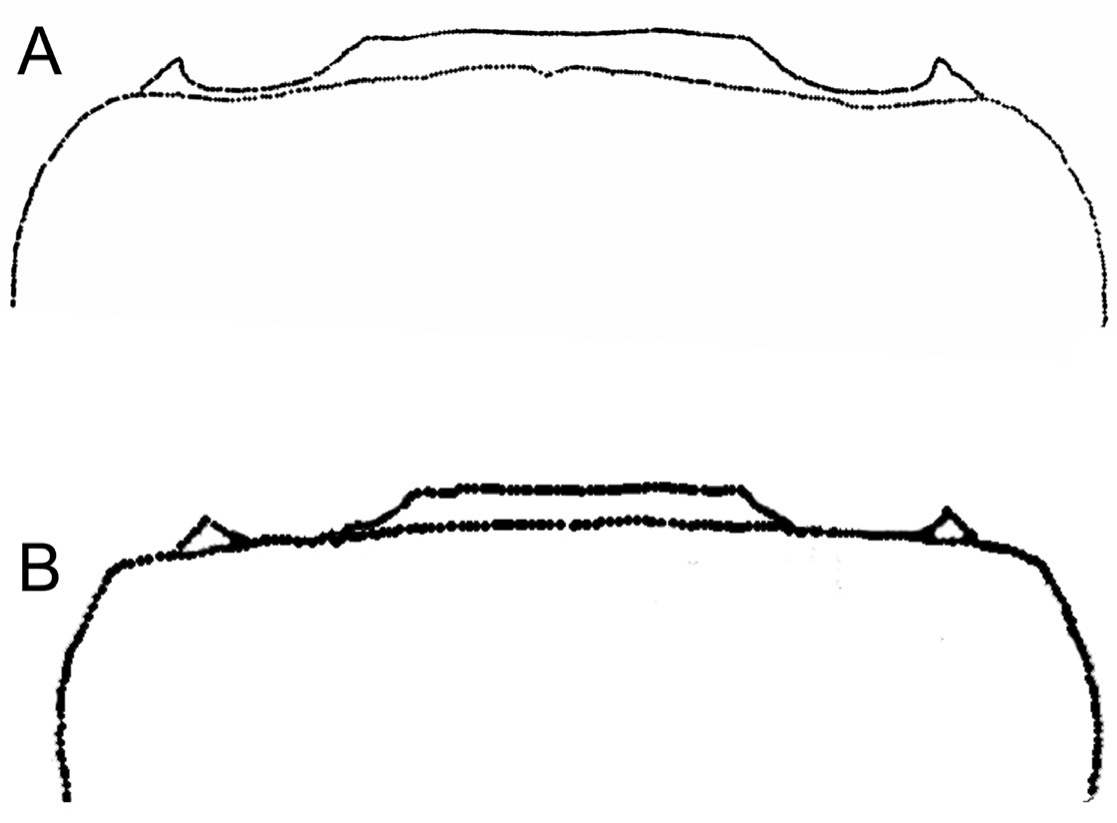
Morphological comparison of carapace features between **A** *Potamonautes perlatus* and **B** *Potamonautes danielsi* sp. n.

*Potamonautes danielsi* sp. n. is easily distinguished from most other South African *Potamonautes* species. *Potamonautes dentatus* Stewart, Coke & Cook, 1995, *Potamonautes parvispina* Stewart, 1997, *Potamonautes unispinus* Stewart & Cook, 1998, *Potamonautes warreni* Calman, 1918 and *Potamonautes calcaratus* (Gordon, 1929) all bear dentate anterolateral margins or epibranchial corners (cf. Stewart et al. 1995: figs 1, 2J; cf. Stewart 1997: figs 2A, 8, 9A; cf. Stewart and Cook 1998: figs 2A, 2D, 6, 7A; cf. Gordon 1929: fig. 1), while *Potamonautes danielsi* has an angular epibranchial corner and slightly scabrous anterolateral margin (Figs 7, 9B).

*Potamonautes clarus* Gouws, Stewart & Coke, 2000, *Potamonautes depressus* (Krauss, 1843), *Potamonautes tuerkayi* Wood & Daniels, 2016, *Potamonautes brincki* (Bott, 1960), *Potamonautes flavusjo* Daniels, Phiri & Bayliss, 2014, *Potamonautes isimangaliso* Peer & Gouws, 2015 and *Potamonautes lividus* Gouws, Stewart & Reavell, 2001 all have smooth anterolateral margins and smooth, rounded epibranchial corners (cf. Gouws et al. 2000: figs 5G, 6, 7A; cf. Krauss 1843: table II fig. 4; cf. Bott 1960: figs 1–4; cf. Daniels et al. 2014: figs 6, 7; cf. Peer et al. 2015: fig. 4; cf. Gouws et al. 2001: figs 5A, 6, 7A), while *Potamonautes danielsi* sp. n. has an angular epibranchial corner and a slightly scabrous anterolateral margin (Figs 7, 9B). Additionally, *Potamonautes danielsi* has a more vaulted carapace (CL/CH = 1.9-2.3) compared to *Potamonautes depressus* (CL/CH = 1.6-1.8), but a flatter carapace compared to *Potamonautes isimangaliso* (CL/CH = 2.3-2.6). *Potamonautes brincki* is confined to the Western Cape. *Potamonautes clarus* and *Potamonautes tuerkayi* are typically bright orange with highly arched dactyls creating a large interspace when chelipeds are closed (cf. Gouws et al. 2000: figs 6A, B, 7C). Conversely, *Potamonautes danielsi* varies in colour from brown to purple but no orange specimens have been collected, while dactyls are moderately arched forming a slim interspace when closed (Figs 7A, 8A, B). *Potamonautes flavusjo* and *Potamonautes lividus* are distinctly coloured (yellow pereopods, and orange pereopods with a blue carapace respectively), with *Potamonautes flavusjo* occurring in the Mpumalanga Highveld and *Potamonautes lividus* mainly in northern KwaZulu-Natal and the Eastern Cape (Gouws et al. 2001, 2015, Daniels et al. 2014), but also in Swaziland (Daniels and Bayliss 2012). *Potamonautes parvicorpus* Daniels, Stewart & Burmeister, 2001 also has rounded epibranchial corners, although it bears a slightly granulated anterolateral margin (cf. Daniels et al. 2001: figs 10, 11A). It also differs in location occurring only in the Western Cape.

*Potamonautes danielsi* shares outward similarities with *Potamonautes perlatus* (H. Milne Edwards, 1837), *Potamonautes granularis* Daniels, Stewart & Gibbons, 1998, *Potamonautes sidneyi* Rathbun, 1904, *Potamonautes barbarai* Phiri & Daniels, 2014, and *Potamonautes barnardi* Phiri & Daniels, 2014. All the above-mentioned species display an angular epibranchial corner with granulation or scabrosity and prominent postfrontal crests (fig. 4A; cf. Daniels et al. 1998: figs 2A, 10, 11A; cf. Rathbun 1904: plate XIV fig. 5; cf. Daniels et al. 1998: fig. 2F). Additionally, these five species are typically widespread, large, robust species occurring from the middle to lower reaches of rivers. *Potamonautes granularis* differs from *Potamonautes danielsi* in that it consistently exhibits orange-tipped chelipeds, the branchial region is highly convex and the anterior margin curves heavily inwards at the midpoint (cf. Daniels et al. 1998: figs 2A, 10, 11A), while *Potamonautes danielsi* does not always have orange-tipped chelipeds, has a flatter branchial region and a fairly straight anterior margin (Fig. 7A). *Potamonautes perlatus*, *Potamonautes barbarai*, and *Potamonautes barnardi* are morphologically indistinct (Phiri and Daniels 2014) and all differ from *Potamonautes danielsi* slightly (Fig. 9). The main difference lies in the anterior margin, which is similar in *Potamonautes sidneyi* *s. str.*, *Potamonautes perlatus*, *Potamonautes barbarai* and *Potamonautes barnardi*. When viewed dorsally, the anterior margin of *Potamonautes danielsi* sp. n. lies relatively straight with a slight forward projection medially (Fig. 9B). The anterior margins of *Potamonautes perlatus*, *Potamonautes barbarai* and *Potamonautes barnardi* all contain a concavity in the crest behind each orbit so that a wide “W” is formed (Fig. 9A). The orbits of *Potamonautes danielsi* are deeper set than the orbits of *Potamonautes perlatus*, *Potamonautes barbarai* and *Potamonautes barnardi*. Additionally, the epibranchial corner of the former is more angular while those of the latter species group are slightly more rounded. However, even here variation across populations makes it difficult to differentiate between these species based solely on morphology.

*Potamonautes danielsi* and *Potamonautes sidneyi* *s. str.* are difficult to distinguish based on morphology alone, as key characters often overlap. The type specimens of the two species exhibit marked differences i.e.: 1) *Potamonautes danielsi* sp. n. has a smoother or slightly granulated anterolateral margin with a smoother or scabrous epibranchial region (fig. 7A), while *Potamonautes sidneyi* *s. str.* typically has a heavily granulated anterolateral margin with a highly scabrous branchial region (plate XIV fig 5-Rathbun, 1904; fig. 4A) and even bears fine hairs on the carapace in some populations; 2) the propodi of *Potamonautes danielsi* sp. n. are inflated (Fig. 8A, B) while those of *Potamonautes sidneyi* *s. str.* are slender (Fig. 5A, B); 3) *Potamonautes danielsi* sp. n. has a high terminal segment length: subterminal segment length of gonopod 2 (Fig. 8H, I) and a low terminal segment length: subterminal segment length of gonopod 1 (Fig. 8F, G), while *Potamonautes sidneyi* *s. str.* has a lower terminal segment length: subterminal segment length of gonopod 2 (Fig. 5H, I) and a higher terminal segment length: subterminal segment length of gonopod 1 (Fig. 5F, G); 4) *Potamonautes danielsi* sp. n. bears slim pereopods (Figs 7A, B) as opposed to the stout limbs of *Potamonautes sidneyi* *s. str.* (Figs 4A, B). However, across the known range of distribution these individual characters vary significantly, with one species resembling the other on occasions. Their separation, thus, requires the inclusion of the whole suite of key characters including the shape and width of the carapace, the inflation of the propodi, the slenderness of the pereopods, the terminal segment length:subterminal segment length ratio of gonopod 1, and the shape of gonopod 1. The granulation of the carapace alone is not a reliable distinguishing character to tell these two species apart. Following morphometric analyses, the new species was distinguished from *Potamonautes sidneyi* *s. str.* mainly by the carapace variables CWA, CL and CH, which contributed the most to distinguishing between the two forms in the discriminant analysis (Fig. 2). The classification functions for both species were as follows:

Y(*Potamonautes danielsi* sp. n.) = 620.17(LogCWA)-1349.21(LogCL)-362.50(LogCH)-382.53

Y(*Potamonautes sidneyi* *s. str.*) = 765.23(LogCWA)-1491.17(LogCL)-305.94(LogCH)-420.01

Individuals were then reassigned to groups based on a priori probabilities, using these classification functions. Ninety-one percent (91%) of the *Potamonautes danielsi* sp. n. individuals and 95% of the *Potamonautes sidneyi* *s. str.* individuals were correctly classified, with only three and one individuals being reassigned to the other species, respectively. The following three regressions were used to support the distinction between the two species: A. P2ML/P2MW, B. CRPW/CRPL, and C. CWA/CL (Fig. 3a, b, c). Regression analyses showed that the two species are significantly distinct using these morphological regressions (P2ML/P2MW-SS = 0.62, df = 1, F = 581, p < 0.001; CRPW/CRPL-SS = 1.11, df = 1, F = 939, p < 0.001; CWA/CL-SS = 0.59, df = 1, F = 2923, p < 0.001).

## Discussion

In a previous study (Gouws et al. 2015), a 9.2–11.8 % divergence was found in the mitochondrial COI and 16S genes of the *Potamonautes sidneyi* clade. Based on the genetic delineation of the two lineages, now *Potamonautes danielsi* sp. n. and *Potamonautes sidneyi* *s. str.*, in Gouws et al. (2015), the distribution of *Potamonautes danielsi* sp. n. is currently known to encompass the coastal zone of southern KwaZulu-Natal, i.e., the northern Pondoland region. However, genetic analyses of recently collected specimens from a wider range of localities (Gouws, unpublished data) suggest a larger distribution of the species. It is likely that it also occurs in Swaziland, but this is yet to be confirmed. With more extensive inland sampling it is possible that the discrete distributions of these species suggested in the earlier study (Gouws et al. 2015) may not be consistent. Furthermore, the huge morphological variation and overlap in key characters obscure purely visual differentiation between these two species in the field. A whole suite of key characters, and possibly even molecular analyses, might be necessary to tell them apart with reasonable confidence.

Phylogenetically, within the southern African potamonautid fauna, *Potamonautes sidneyi* *s. str.* belongs to the clade of large-bodied, robust freshwater crabs, including *Potamonautes perlatus*, *Potamonautes granularis*, *Potamonautes barbarai* and *Potamonautes barnardi* (Daniels et al. 2002, 2015), that are mostly confined to the middle to lower reaches of rivers (Daniels et al. 2002). The derivation of these species from a common ancestor explains their overall morphological similarities. *Potamonautes danielsi*, however, appears to belong to a clade that includes burrowing species such as *Potamonautes isimangaliso*, *Potamonautes lividus* and *Potamonautes flavusjo* (Gouws et al. 2015), all three of which are easily distinguishable from *Potamonautes danielsi*. While the phylogenetic placement of these taxa requires more rigorous testing, it would appear that the morphological similarity between *Potamonautes danielsi* sp. n. and *Potamonautes sidneyi* *s. str.* is not phylogenetically determined and may well reflect habitat similarities and environmental drivers, given their current distributions. This may support the lack of distinctive species-specific characters that appears to be a widespread trend for African potamonautids (Daniels et al. 2003, Jesse et al. 2010, Cumberlidge and Daniels 2014, Phiri and Daniels 2014, Phiri and Daniels 2016).

South Africa is fairly well-studied regarding the taxonomy of freshwater crab fauna (Daniels et al. 2014, Phiri and Daniels 2016) and yet ongoing molecular work still yields novel undescribed species of *Potamonautes*, many of which are cryptic and thus easily mistaken for previously recorded species in the past (Phiri and Daniels 2014). The large number of freshwater habitat types present through South Africa, ranging from the headwaters of the Drakensberg region to coastal freshwater seepage points, is associated with the high diversity and endemism of freshwater brachyurans found in the country (Darwall et al. 2009). Some species appear to be quite adaptable, inhabiting a variety of habitat types, such as *Potamonautes perlatus* which extends from the inland region of the Northern Cape Province to freshwater seepage barrage pools along the Eastern Cape coast (Perissinotto et al. 2014), withstanding a wide range of altitude, salinity and temperature throughout its distribution. Conversely, certain species occupy very specific niches, such as *Potamonautes isimangaliso* which is currently only known from ephemeral pans along the western shore of False Bay, iSimangaliso Wetland Park (Peer et al. 2015a).

**Figure 10.**
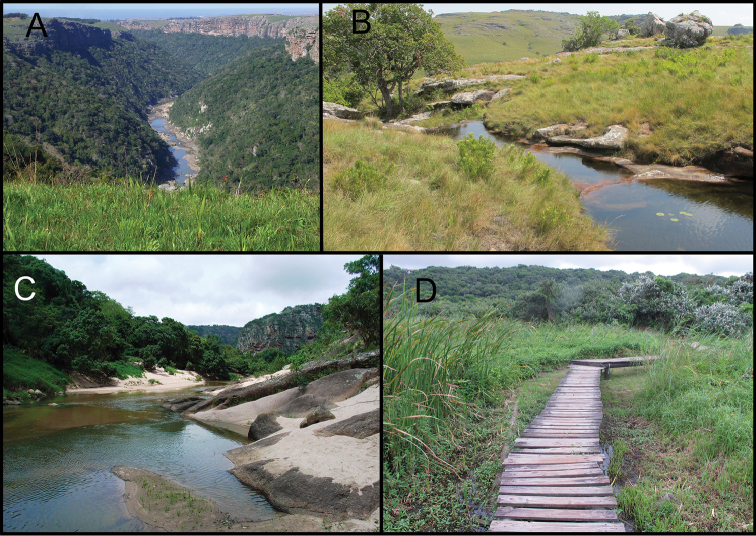
Habitat types of *Potamonautes danielsi* sp. n. Various habitat types of *Potamonautes danielsi* sp. n. **A** and **B** Mtamvuna Nature Reserve showing the dense canopy cover under which specimens were collected as well as open streams and pools **C** Oribi Gorge Nature Reserve, and **D** Umhlanga Nature Reserve. Photos: A, B, C-Lynette Clennell; D-Nasreen Peer.

*Potamonautes danielsi* sp. n. does not appear to be habitat-specific but seems to prefer purely freshwater habitats established in areas with summer rainfall (Fig. 10). In Mtamvuna and Oribi Gorge, specimens were found under boulders and logs in mountain streams (altitude = 140-150 m) flowing into or connected to the main rivers. The Umhlanga Reserve consists mostly of KwaZulu-Natal coastal belt (CB3 vegetation unit-Mucina and Rutherford 2006) with a small portion of Northern Coastal forest (FOz7 vegetation unit). While the latter is classified as least threatened, its location next to an endangered CB3 habitat type within a growing urban area means that the surrounding habitat is already heavily transformed. The crab populations in this reserve were found close to sea level in the coastal belt habitat, burrowing under dominant grasses and shrubs near a freshwater seepage area which is connected to the Mhlanga River Mouth. Both the Mtamvuna and Oribi Gorge reserves consist of Scarp Forest (FOz 5), which is well-known for housing many endemic tree species and forms a core component of the Pondoland Centre of Endemism (van Wyk and Smith 2001). These forests exhibit tall and well-developed canopy and understorey tree layers that provide moist damp areas during the rainfall season (spring-summer), with adequate shade and shelter for the crab populations dwelling in the streams.

The species is sympatric with *Chiromantes eulimene* (de Man in Weber, 1898) in all three habitats, although the latter prefers the reed-like habitat adjacent to the main river, while *Potamonautes danielsi* appears to prefer the slower-flowing streams running into the main river body.

A greater number of *Potamonautes danielsi* adults were found under boulders and detritus in the water, as opposed to in burrows, although no feeding behaviour was observed at the time of collection. Generally, the feeding ecology of all *Potamonautes* spp. is supposedly opportunistic and thought to shift with age. Gut content analyses and stable isotope analyses have been conducted on *Potamonautes perlatus* (see Hill and O’Keeffe 1992) and *Potamonautes sidneyi* (see Peer et al. 2015b), respectively. While adults of these two species are mainly herbivorous and detritivorous, juveniles appear to favour a carnivorous diet. This could possibly relate to the ontogenetic shift in habitats, where juveniles occupy the water body, while adults reside in burrows on the banks of streams or rivers. Juveniles encounter more potential prey items on the benthos than adults do near their burrows. Thus, the presence of *Potamonautes danielsi* adults in the water body means that a wider range of prey is available to them for consumption. However, the overall ecology of the southern African potamonautids remains highly understudied. As new species are described, interspecific ecological differences are becoming apparent highlighting the need for more ecological research in this field.

Currently, 20 species of *Potamonautes* have been described in South Africa with six additional new but undescribed species (Phiri and Daniels 2016). Daniels et al. (2014) highlighted the relatively poor exploration of high altitude mountainous freshwater habitats and predicted that future collections from these understudied areas will yield new undescribed species. This sheds new light on inland freshwater habitats, in terms of conservation, as highly endemic or specialist species are often a priority in earmarking areas for protection. As recent national biodiversity assessments regarding South Africa’s inland water systems have highlighted the threat faced by these largely unprotected systems (Nel et al. 2011), exploring and documenting their rich biodiversity and specialised ecology should be prioritised.

## Supplementary Material

XML Treatment for Potamonautes sidneyi

XML Treatment for Potamonautes danielsi
